# Neonatal Deafening Selectively Degrades the Sensitivity to Interaural Time Differences of Electrical Stimuli in Low-Frequency Pathways in Rats

**DOI:** 10.1523/ENEURO.0437-22.2022

**Published:** 2023-01-17

**Authors:** Woongsang Sunwoo

**Affiliations:** Department of Otorhinolaryngology, Gil Medical Center, Gachon University College of Medicine, Incheon 21565, Republic of Korea

**Keywords:** auditory deprivation, binaural hearing, cochlear implant, development, inferior colliculus, interaural time difference

## Abstract

We examined the effect of neonatal deafening on frequency-specific pathways for processing of interaural time differences (ITDs) in cochlear-implant stimuli. Animal studies have demonstrated differences in neural ITD sensitivity in the inferior colliculus (IC) depending on the intracochlear location of intracochlear stimulating electrodes. We used neonatally deafened (ND) rats of both sexes and recorded the responses of single neurons in the IC to electrical stimuli with ITDs delivered to the apical or basal cochlea and compared them with acutely deafened (AD) rats of both sexes with normal hearing (NH) during development. We found that neonatal deafness significantly impacted the ITD sensitivity and the ITD tuning patterns restricted to apically driven IC neurons. In ND rats, the ITD sensitivity of apically driven neurons is reduced to values similar to basally driven neurons. The prevalence of ITD-sensitive apical neurons with a peak-shaped ITD tuning curve, which may reflect predominant input from the medial superior olivary (MSO) complex, in ND rats was diminished compared with that in AD rats (67%, AD vs 40%, ND). Conversely, monotonic-type responses rarely occurred in AD rats (14%) but were approximately equally as prevalent as peak-type tuning curves in ND rats (42%). Nevertheless, in ND rats, the ITD at the maximum slope of the ITD tuning curve was still more concentrated within the physiological ITD range in apically driven than in basally driven neurons. These results indicate that the development of high ITD sensitivity processed by low-frequency pathways depends on normal auditory experience and associated biases in ITD tuning strategies.

## Significance Statement

Binaural neurons in the inferior colliculus (IC) are sensitive to interaural time differences (ITDs) with a high resolution when electrical pulse trains are presented through a low-frequency pathway compared with a high-frequency pathway. However, despite the potential importance of intracochlear stimulating sites in improving ITD sensitivity, it is unclear whether these functionally segregated pathways can be preserved following early auditory deprivation. Here, we show that site-specific ITD circuitry from the cochlea to the IC can be partly developed without auditory experience in neonatally deafened animals. This finding may support the importance of selective electrical stimulation of the apical region of the cochlea for achieving improved sensitivity to ITDs in bilateral cochlear implant (CI) users, even after early auditory deprivation.

## Introduction

Cochlear implants (CIs) are typically recommended for bilaterally hearing-impaired patients who have limited benefit from a conventional hearing aid and substantially reduced speech recognition ability even in the aided condition (Varadarajan et al., 2021). Two CIs can be implanted bilaterally, sequentially and simultaneously in a single surgery; the latter option is favored in children because of childhood being a sensitive period for the development of the central auditory system (Papsin and Gordon, 2008; Uecker et al., 2019; Gao et al., 2020). Bilateral CIs have been proven to carry binaural benefits, such as improved sound localization and speech perception in noise (Nopp et al., 2004; Grantham et al., 2007; Zeitler et al., 2008; Eapen et al., 2009; Lovett et al., 2010).

To fully use binaural benefits from bilateral implantation, the ability to detect the interaural time difference (ITD) is required to be as sensitive as normal hearing (NH) listeners. However, previous studies have revealed that bilateral CI users show limited sensitivity to ITDs compared with NH listeners, even with research processors that can provide ITD information optimally (Litovsky et al., 2012; Laback et al., 2015). One of the potential factors contributing to the limitations of ITD sensitivity in bilateral CI users is early auditory deprivation during developmental periods. For example, poor ITD sensitivity is particularly observed in prelingually deafened bilateral CI users without a normal hearing experience (Litovsky et al., 2010; Easwar et al., 2017; Ehlers et al., 2017). The effects of early auditory deprivation on ITD sensitivity are also supported by physiological data from neonatally deafened animals (Hancock et al., 2010, 2013; Tillein et al., 2010; Chung et al., 2019; Thompson et al., 2021). Likewise, anatomic studies of the medial superior olive (MSO), which is the primary auditory nucleus specialized to extract ITDs, indicate that auditory experience is important for the refinement and reorganization of developing brainstem circuits that are relevant for the precise processing of ITDs (Kapfer et al., 2002; Werthat et al., 2008).

Because the use of ITD cues critically depends on the sound frequency in NH listeners (Wightman and Kistler, 1992; Macpherson and Middlebrooks, 2002), the dependence of ITD sensitivity on the tonotopic location of electrical stimulation has been hypothesized in bilateral CI users. However, psychophysical studies have reported no significant effect of electrode location on the ITD thresholds (Laback et al., 2015; Thakkar et al., 2020). A possible reason for these findings might be inadequate stimulation of the low-frequency pathway because of limitations in the insertion depth of commercially available electrode arrays, which mostly cover a frequency range of only up to ∼1000 Hz or higher (Dhanasingh and Jolly, 2017). Recently, a physiological study using direct electrode positioning through cochleostomies to provide selective stimulation of frequency-specific pathways demonstrated differences in neural ITD sensitivity in the inferior colliculus (IC) depending on the tonotopic location of intracochlear stimulation (Sunwoo and Oh, 2022). This work suggests that binaural neurons in the IC are sensitive to ITDs with a high resolution when electrical pulse trains are presented through low-frequency pathways compared with high-frequency pathways. However, despite the potential importance of intracochlear stimulating sites in improving ITD sensitivity, it is unclear whether these observations can be preserved following early auditory deprivation. Thus, a better understanding of whether and to what degree these central mechanisms involved in ITD processing are altered by the lack of auditory experience in neonatally deafened animal models could help improve the performance of prelingually deaf CI users. To examine this issue, we deafened rats neonatally with ototoxic drugs and recorded the responses of single neurons in the IC to electrical pulse trains with ITDs delivered to the apical or basal cochlea and compared them with previous data from acutely deafened rats as an adult which had normal hearing experience during development.

## Materials and Methods

### Animals and experimental design

Breeding pairs of Wistar-Albino rats were obtained from Orient Bio Inc. (Seongnam). Then, rat pups were bred and raised to early adulthood (10–12 weeks) under standard housing conditions (food and water *ad libitum*; 12/12 h light/dark cycle; temperature 22 ± 2°C; humidity 40–60%). Twenty-five rats (13 males and 12 females) were deafened as neonates and implanted at 10–12 weeks of age. Neurophysiological recording from the central nucleus of the IC was performed in all neonatally deafened rats (hereinafter referred to as the “ND” group, *n* = 25). Neural information from the ND group was compared with reanalyzed neural data from 27 rats of both sexes that were acutely deafened as adults on the same day as they were implanted at 12 weeks of age (hereinafter referred to as the “AD” group, *n* = 27; Sunwoo and Oh, 2022). Animals in the AD group had normal hearing development and experienced normal binaural hearing until the day of the electrophysiological recording. All experimental and animal care procedures were approved by the Institutional Animal Care and Use Committee at the Gachon University Gil Medical Center (MRI-2020–0008-1) and performed in accordance with institutional guidelines.

### Anesthesia

Animals were anesthetized by intraperitoneal injection of a mixture of ketamine hydrochloride (80 mg/kg) and xylazine hydrochloride (10 mg/kg) for physiological recording procedures. To maintain the animal at the appropriate anesthetic plane indicated by no toe-pinch reflex, a one-third dose of the original drug combination was administered every ∼45 min throughout the experiments. For surgical procedures, anesthesia was achieved with low-dose isoflurane (1–3%) inhalation, which has been found to reduce perioperative mortality compared with injectable anesthesia (Claussen et al., 2019). After surgery, the administration of isoflurane was discontinued, and the anesthetic was carefully changed to a ketamine/xylazine mixture for recordings because ketamine-based anesthesia affects hearing sensitivity less compared with isoflurane anesthesia (Cederholm et al., 2012). During anesthesia, body temperature was monitored using a rectal probe and maintained using a heating pad.

### Deafening procedure

Except for the littermates used as controls in this investigation, all animals were ND. From the eighth day after birth, daily intraperitoneal injections of kanamycin solution (Sigma-Aldrich; 50 mg/ml in 0.9% NaCl) at a dosage of 400 mg/kg were administered for a total of 14 d (Osako et al., 1979; Onejeme and Khan, 1984). On the day after the end of the antibiotic injection, auditory brainstem responses (ABR) to clicks were recorded on both sides in a soundproof chamber. For ABR recording, the Tucker-Davis Technologies (TDT) System three hardware devices (RZ6 processor and Medusa4Z preamplifier) and the BioSigRP software (TDT) were used. Three subdermal electrodes were inserted at the vertex and both mastoids. A 20-Hz train of click (0.1-ms) stimuli was presented in a closed field by an electrostatic speaker (EC1; TDT) coupled to a Tygon tube with an ear tip inserted into the ear canal. Click levels varied from 80 to 5 dB SPL in 5-dB decrements. ABR data, bandpass filtered from 300 to 3000 Hz, were obtained 500 times and averaged. The presence of an identifiable waveform to clicks delivered at the maximum intensity tested (90 dB SPL) indicated incompletely deafened animals that may have had auditory experience. Consequently, the rats showing click responses (≤90 dB) after the deafening procedure were not used further in the study. After exclusion, 25 experimental animals (13 males and 12 females) were included.

### Intracochlear implantation and craniotomy

**Figure 1.**
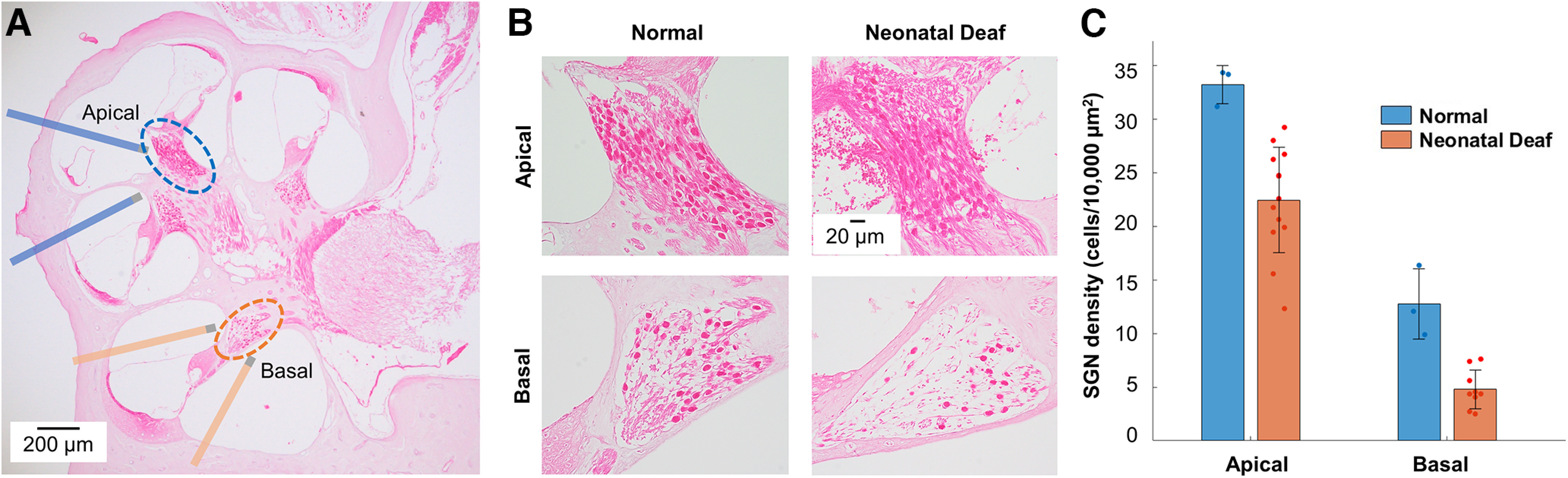
***A***, Representative image of a hematoxylin and eosin stained midmodiolar section from a rat cochlea showing the two locations indicated by the dashed lines at which the spiral ganglion neurons (SGNs) were stimulated by the apical and basal bipolar electrode pairs, respectively (20×). ***B***, Representative images of the SGNs in the apical and basal turns for the normal controls and neonatally deafened (ND) animals (40×). ***C***, Mean SGN densities at two different cochlear locations for the normal controls and ND animals. Error bars represent ±1 SD.

At 10–12 weeks of age (weights: 487.7 ± 59.4 and 304.9 ± 25.8 g in male and female rats, respectively), ND rats were bilaterally implanted with four polytetrafluoroethylene insulated 90% platinum-10% iridium wires (A-M Systems, 127 μm in bare diameter) with uncoated tips over 250 μm on each side, using previously published techniques (Sunwoo and Oh, 2022). In brief, single stimulating electrodes were placed close to the modiolus one by one through four holes made in the cochlear bone. Consequently, the apical bipolar pair of two electrodes (one in the apical scala tympani and one in the middle scala vestibuli) was intended to selectively stimulate the cochlear fibers corresponding to low frequencies, whereas the basal bipolar electrode pair (one in the basal scala tympani and one in the basal scala vestibuli) was devoted to the high-frequency cochlear fibers (Fig. 1*A*). In a previous study, rats were implanted unilaterally, and the frequency response area analysis determined the characteristic frequencies for each electrode pair using auditory stimulation of the intact ear; ∼2.5 kHz for the apical pair and 21.6 kHz for the basal pair (Sunwoo and Oh, 2022). Implanted intracochlear electrodes were secured inside the bulla using a self-adhesive resin cement (GC Corp.).

Following bilateral cochlear implantation, small craniotomies were made in both parietal bones to allow the insertion of a recording electrode into the IC. Based on stereotaxic coordinates, an opening ∼5 mm in diameter was positioned 2–5 mm lateral and 0.3–0.5 mm rostral to λ (Paxinos and Watson, 2007). The exposed dura was preserved to prevent cerebrospinal fluid from leaking and covered with 0.9% saline to prevent the cortical surface from drying during the recording procedures.

### Electrophysiological data acquisition

To check for malfunction or displacement of the implanted electrodes, electrically evoked ABRs (EABRs) were tested before data collection from the IC. Immediately after surgery, EABRs were recorded for each stimulating electrode pair in response to bipolar electrical stimulation. The stimuli were charge-balanced biphasic current pulses with a leading cathodic phase (40 μs) immediately followed by an anodic phase (40 μs), delivered at 50 pulses per second (pps). The current was increased in steps of 2 dB relative to (re) 1 mA from −20 to 0 dB (corresponding to 100 μA to 1 mA). In this study, all implanted electrodes were demonstrated to be effective for intracochlear electrical stimulation by measuring the EABR thresholds below −10 dB re 1 mA (apical pair, median 126 μA and range 100–316 μA; basal pair: median 316 μA and range 126–316 μA).

To improve the signal-to-noise ratio, extracellular recordings were performed with the rats fixed with a stereotaxic instrument inside the Faraday cage mounted on the vibration isolation table. The recording probe had 16 channels, 177 mm^2^ in area, spaced at 100-μm intervals along a single silicon-substrate shank (NeuroNexus), and was advanced into the IC through the occipital cortex, either in a vertical or oblique penetration, using a motorized micromanipulator (Scientifica). A reference wire (Ag/AgCl) with a recording probe was inserted into the craniotomy contralateral to the recording probe and was placed on the intact dura. Neural activities acquired by these 16-channel electrodes were amplified, filtered (bandpass, 300–5000 Hz), and digitized (sampling rate, 24.41 kHz) using the TDT system comprising a ZC16 analog headstage, SIM neurodigitizer, and RZ5P signal processors (TDT). Digital data were then monitored and processed online to extract neural spike events using Synapse software (TDT).

While slowly lowering the recording probe into the brain, a series of three biphasic current pulses (cathodic leading, 40 μs per phase) with a 100-ms interval was delivered to search for IC neurons. The intensity of the search stimuli was identical in both ears and was usually 4–6 dB above the measured EABR thresholds. Responsive neurons were first identified by apical pair stimulation in the dorsal IC, followed by basal pair stimulation in the ventral IC. After a single IC neuron was isolated by micromanipulation, which was indicated by its constant spike waveform and demonstrated by grouping into only one cluster when using a real-time principal component-based spike sorter in the Synapse software (TDT), search stimuli with variable intensity (from −20 to 0 dB re 1 mA, 2-dB step) were presented to determine each neuron’s thresholds and the current level for subsequent experiments (usually 4 dB above the threshold for diotic stimulation). In addition, the time between successive spontaneous spikes was measured offline as the second criterion for the single-unit recording. If <1% of interspike intervals were <2.5 ms, the recording was considered a single-unit activity. In a previous study of AD animals, none of the IC neurons showed binaural responses to both apical and basal pair stimulations at current levels of <1 mA (Sunwoo and Oh, 2022). Similarly, in the present study using ND animals, IC units in the dorsal region responding to the apical pair were not stimulated by the basal pair, even at levels up to 1 mA. However, some IC units in the ventral region not only responded to the basal pair stimulation but also the stimulation of the apical pair when a higher intensity was applied, which is presumed to be the cochleotopic reorganization in the IC by neonatal deafening rather than intracochlear electrical current spread. Because we aimed to compare the effects of stimulation sites on ITD sensitivity between the AD and ND groups, further data were acquired only from the well-isolated single IC neurons that responded to only one stimulating electrode pair, either the apical or basal, similar to a previous study.

Then, 300-ms pulse trains with a 1000-ms repetition time period, variable pulse rate, and ITD were presented binaurally to study the neuron’s ITD sensitivity. The pulse rates ranged from 20 to 1280 pps in one-octave steps, and the ITDs ranged from −1200 to 1200 μs in 200-μs steps. When a stimulus was led in the cochlea contralateral (or ipsilateral) to the recording IC, the ITD values were documented as positive (or negative, relatively). For a given stimulation condition, each pulse train was presented in a pseudo-random order and repeated ten times in total.

### Electrophysiological data analysis

The methods used to analyze neural ITD sensitivity were the same as those previously described (Chung et al., 2019; Sunwoo et al., 2021; Sunwoo and Oh, 2022). ITD sensitivity analyses were performed offline on the neural firing rates extracted from the digitized recordings. The average number of spikes across all trials of the same stimulus condition over the entire 300-ms duration of pulse trains was used to compute the “total” firing rate. In addition, we also computed neural firing rates particularly based on “sustained” responses which occurred for 280 ms after the first 20 ms of stimulus. The statistical analyses of “onset” responses have very low statistical power because of a low number of stimulus repetitions and are not reported. To assess the sensitivity of neurons to ITD, we used two metrics based on the signal-to-total variance ratio (STVR) and just noticeable difference (JND; Chung et al., 2016). The ITD STVR is based on the ANOVA metric and is defined as the fraction of the effect variance in firing rates in response to the varying ITD to the total variance in firing rates across all trials. By definition, the STVR can have values between 0 (not sensitive to the varying ITD) and 1 (neural activity changes only with variations in ITD). The statistical significance of ITD sensitivity based on the STVR metric was determined using an *F* test (*p *<* *0.01).

**Figure 2.**
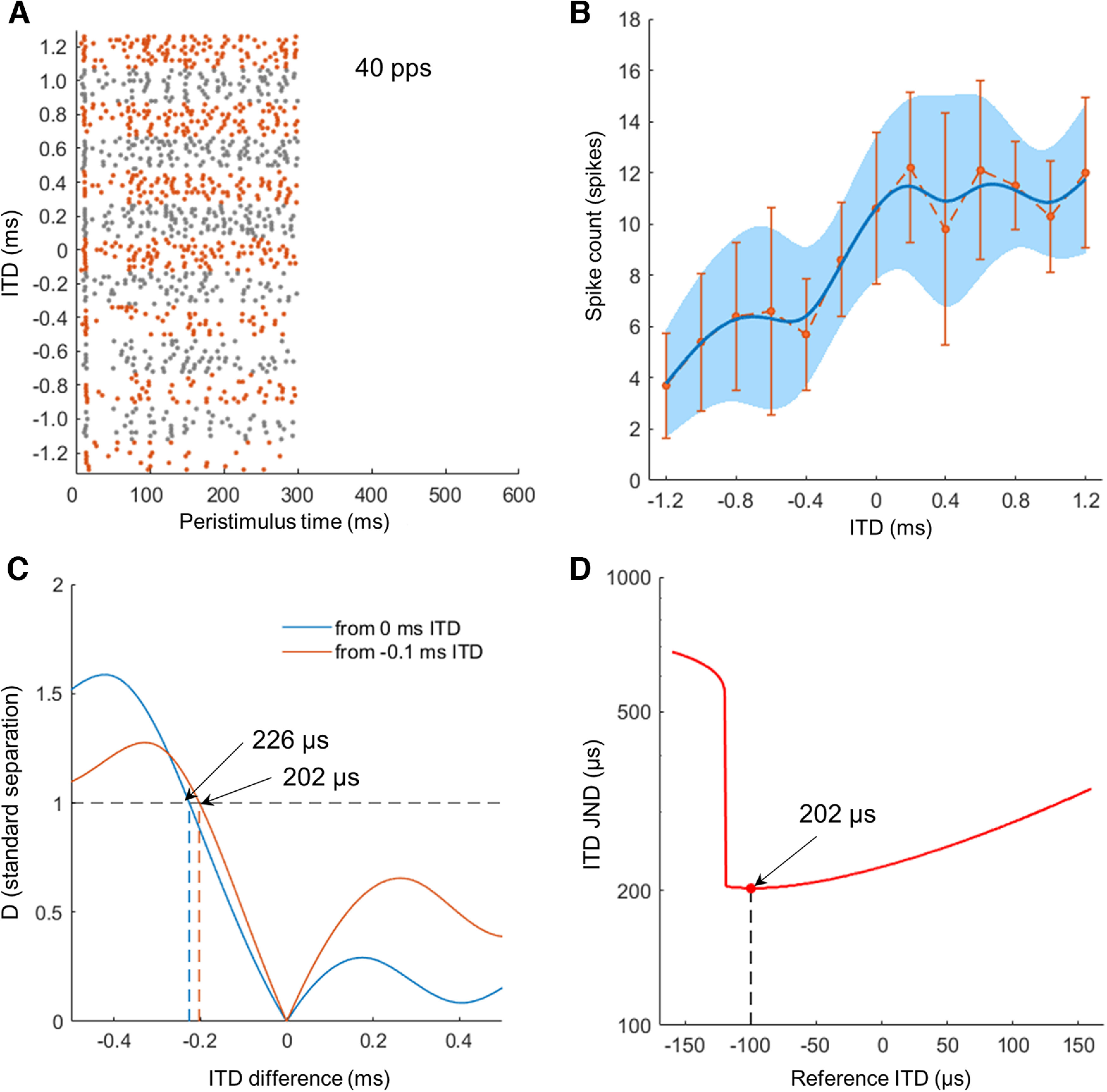
***A***, Temporal discharge pattern (dot rasters) of an example neuron for 40-pps pulse trains as a function of the interaural time difference (ITD). ***B***, The mean spike count for the entire stimulus duration of 300 ms as a function of the ITD (dashed line). Error bars indicate ±1 SD. To smooth out noise in ITD tuning curve, both the mean (thick blue line) and the variance (light blue shade) of the spike count were fitted with a cubic spline. ***C***, Standard separation (D) curves for two different reference ITD (blue line for 0 μs and orange line for −100 μs) as a function of ΔITD (difference from a reference ITD). ITD JNDs (arrow) at each reference ITD are where D = 1 (black dashed horizontal line). ***D***, Neural ITD JND curve as a function of a reference ITD within the physiological range of ITD (±160 μs). The smallest value of 202 μs is determined as the ITD JND of an example neuron.

In the other metric, as an estimate of ITD discrimination thresholds of single IC neurons, the ITD JND was obtained from spline-fitted curves of both the mean and the variance of the spike count as a function of ITD (Fig. 2*A*,*B*) using the modified version of the standard separation, which is computed as the ratio of the difference in the mean spike counts at two different ITDs divided by the square root of the arithmetic mean of variances in spike counts as follows (Sakitt, 1973; Smith and Delgutte, 2007):

$$D_{\text{ITD},\text{ ITD}+\Delta \text{ITD}}=\frac{\left|\mu_{\text{ITD}}-\mu_{\text{ITD}+\Delta \text{ITD}}\right|}{\sqrt{(\sigma^{2}{}_{\text{ITD}}+\sigma^{2}{}_{\text{ITD}+\Delta \text{ITD}})/2}},$$

where $\mu_{\text{ITD}}$ and $\sigma^{2}{}_{\text{ITD}}$ are the mean spike counts and the respective variance at the reference ITD, and ΔITD is the difference between the test ITD and the reference ITD. To optimize the reference ITD and minimize the JND (Smith and Delgutte, 2007), the standard separation curve as a function of ΔITD was computed for all physiologically possible reference ITDs in rats ranging from −160 μs to 160 μs (Koka et al., 2008; Fig. 2*C*). The smallest ITD difference, with an absolute standard separation value of 1, was determined as the ITD JND of each reference ITD. Then, ITD JND curve as a function of reference ITD can be constructed and the smallest value was determined as the ITD JND of each neuron (Fig. 2*D*). If a neuron did not show a standard separation value of one or greater within the range of ΔITDs tested from any of reference ITDs (±160 μs), the ITD JND was reported to be unmeasurable.

When neurons showed a significant ITD-sensitive response with STVR values above zero to pulse trains with pulse rates below 320 pps, they were included in further analysis of the ITD tuning curves. In each ITD-sensitive unit, the responses to the pulse rate that maximized the ITD STVR were processed to construct the ITD tuning curve (average firing rate as a function of ITD). The ITD tuning curve could become periodic at high pulse rates of 640 and 1280 pps, within the ±1200-μs range of ITDs tested, which prevents the appropriate classification of ITD tuning shapes. When the absolute value of ITD corresponds to half of the period of the pulse train, the contralateral and ipsilateral leading stimuli become identical. Although it is not exactly a periodic curve at 320 pps, the extreme ITDs on both sides are very close to half of the period (1562.5 μs). Because the ITD tuning curves to 320-pps pulse trains have similar firing rates at both ends, it cannot be the best fit with a sigmoid function. Then, we classified the shapes of the ITD tuning curves into four types, including peak, trough, monotonic, and unclassified, by a template fitting method using the sum of the Gaussian and sigmoid functions, as suggested by Chung et al. (2016):

$$Rate\left(ITD\right)=A*2^{-{\left(\frac{ITD-B}{C/2}\right)}^{2}}+\frac{D}{1+3^{-2\left(\frac{ITD-B}{C}\right)}}+E,$$

where *A*, *D*, and *E* represent the scaling factors, and *B* and *C* represent the center and half-width (or half-rise) of the Gaussian and sigmoid functions, respectively. After fitting the data using the Curve Fitting Toolbox software (The MathWorks), the goodness of fit was evaluated by the *R*^2^ value. In cases with an *R*^2^ value of 0.75 or higher, the fitted curves were classified into the peak and trough types by the presence of the central maximum and minimum, or into the monotonic type. In detail, the peak-type (or the trough-type) curve was defined as a curve in which the firing rate decreased (or increased) by 20% of the difference between the maximum and minimum value of the given curve from the central maximum (or minimum) on both sides. Otherwise, the firing rate decreased from the maximum value over 20% of the range on only one side, which was classified as the monotonic-type curve. If the ITD tuning curves were considered unsuitable for fitting to the template (*r*^2^ < 0.75) and were grouped as “unclassified.” Then, to investigate how the ITD tuning curve type distributions influence the ITD sensitivity, the ITD at the steepest or maximum slope (ITD_MS_), where ITD discrimination is maximal, was derived from the well-fitted tuning curve.

### Histologic analysis

Following the neurophysiological experiments, 16 cochleae from eight ND rats were harvested. To provide control data for comparison, a total of three cochleae from the two control littermates underwent the same histologic procedure. Before transcardial perfusion, the animals have heavily anesthetized with an additional dose of ketamine (80 mg/kg)/xylazine (10 mg/kg) mixture. Afterward, they were immediately transcardially perfused with phosphate-buffered saline (pH 7.4), followed by 10% neutral buffered formalin. Then, the temporal bones were dissected from the head and placed in the same fixative for cochlear perfusion through the scalae. After overnight postfixation, the cochleae were decalcified using a decalcifying agent (Calci-Clear Rapid; National Diagnostics). Decalcified tissues were dehydrated and embedded in paraffin blocks. The temporal bones were sectioned on the perimodiolar plane using a rotary microtome (Leica RM2255) at a thickness of 5 μm. Every fifth section was mounted on a coated glass slide and stained with hematoxylin and eosin.

For each cochlea, five mid-modiolar sections were selected for the quantitative analysis of intact spiral ganglion neurons (SGNs). In this study, two regions of interest were analyzed for each section: Rosenthal’s canal in the apical and basal turns of the cochlea (Fig. 1*B*). The sections were examined, and digital images were acquired using a light microscope equipped with a digital camera (Olympus BX53/DP74). The SGN count and the area within the bony boundaries of Rosenthal’s canal were measured using Olympus cellSens software (RRID:SCR_014551). The density of SGN was calculated by dividing the number of counted SGN by the area of Rosenthal’s canal. The SGN densities from three to five representative sections were then averaged to obtain one value per cochlear location.

### Statistical analysis

The data analysis and statistical tests were performed using IBM SPSS Statistics (IBM Corp.), MATLAB (The MathWorks), and R 4.1.1 (R Project for Statistical Computing).

Categorical data were analyzed using the χ^2^ test and Fisher’s exact test. To compare the percentages of neurons showing significant ITD sensitivity as a function of pulse rate between the two stimulation positions, two-way analysis of deviance with binomial generalized linear mixed models (GLMMs) and *post hoc* pairwise comparisons were run on the binary variable for the absence/presence of ITD sensitivity. To approximate the normality assumption of ANOVA, ITD STVRs were transformed using logit transformation. For the effect of the location of stimulation, pulse rate, and their interaction on the transformed ITD STVR, a two-way ANOVA with linear mixed models (LMMs) was used. *Post hoc* pairwise comparisons with Bonferroni’s correction were performed for data with a significant ANOVA effect. To determine the relative contribution of the sustained response to the degree of ITD sensitivity between the two stimulation positions at different pulse rates, a two-way ANOVA with LMMs on the logit-transformed STVR based on the sustained response was used. To estimate differences in mean 25th percentile ITD JNDs between apical and basal units at each pulse rate, a set of 10,000 bootstrap trials was analyzed. Differences in ITD_MS_ distributions between the apical and basal units were tested using Levene’s test. Statistical significance was set at *p *<* *0.05.

## Results

This study included single-unit recordings of 100 IC neurons in 25 ND rats of both sexes. The number of recorded units per animal ranged between two and eight, with a mean of 4.0 ± 1.8 (SD) and a median of four. Sixty-three (63%) of these neurons were assigned to the apical units and 37 (37%) to the basal units because they were selectively activated by stimuli from the electrode pair located in the apical and basal cochlea, respectively. It should be noted that neurons responsive to both apical and basal stimuli, which could not be assigned unambiguously to either the apical or basal units, were excluded. Neural information was also compared with previously reported data from 108 IC neurons, including 53 (49%) apical units and 55 (51%) basal units, in 27 AD rats (Sunwoo and Oh, 2022).

To investigate the morphologic changes in the auditory periphery induced by the neonatal deafening procedure, we compared the density of SGN in normal cochleae and electrode-implanted cochleae of ND rats. Figure 1*B* shows representative examples of SGN survival in the apical and basal turns on H&E staining of the cochlear sections of AD and ND rats. It is evident from the images in Figure 1*B* that neonatal deafening leads to more severe degeneration of the SGN in the basal turn than in the apical turn. Figure 1*C* summarizes the quantitative data for SGN density in each cochlea in both normal controls and ND rats. The data obtained from two littermate controls with normal hearing were averaged and used as a normative reference to express SGN survival as a percentage of the normal SGN density in this study. Although the survival of SGNs in the AD group was not measured previously, the AD group, which was deafened just before the neurophysiological experiments, was considered to have normal or nearly normal SGNs. Furthermore, it has been demonstrated that acute intracochlear electrical stimulation, even with a high intensity of 6 dB above the electrically evoked compound action potential threshold for 4 h, did not induce significant structural changes in SGNs (Q. Li et al., 2020). In the ND group, the average SGN density in the apical regions was 67.6% of normal, whereas SGN survival in the basal regions was reduced to 37.5% of normal. The Wilcoxon signed-rank test indicated that, compared with the average value of normal controls, the SGN density reduction rate in the ND group was greater in the basal SGNs (median 65.8%, range 40.4–80.4%) than the apical SGNs (median 32.0%, range 12.0–62.9%; *T *=* *36, *z* = −2.67, *p *= 0.008). In addition, to determine whether there was a mean difference in the area of Rosenthal’s canal itself between normal controls and animals who underwent a neonatal deafening procedure, a *t* test was conducted on all mid-modiolar sections used for SGN density calculation. There was no significant difference in the apical area for normal (3.27 ± 1.09 × 10^4^ μm^2^) and ND (3.21 ± 0.59 × 10^4^ μm^2^) conditions (*t*_(41.305)_ = 0.292, *p *=* *0.772, two-tailed). Similarly, there was no significant difference in the area of basal Rosenthal’s canal between normal (3.92 ± 0.78 × 10^4^ μm^2^) and ND animals (3.90 ± 1.07 × 10^4^ μm^2^; *t*_(58)_ = 0.056, *p *= 0.956, two-tailed). These results suggest that neonatal deafening procedure does have a detrimental effect on SGNs.

**Figure 3.**
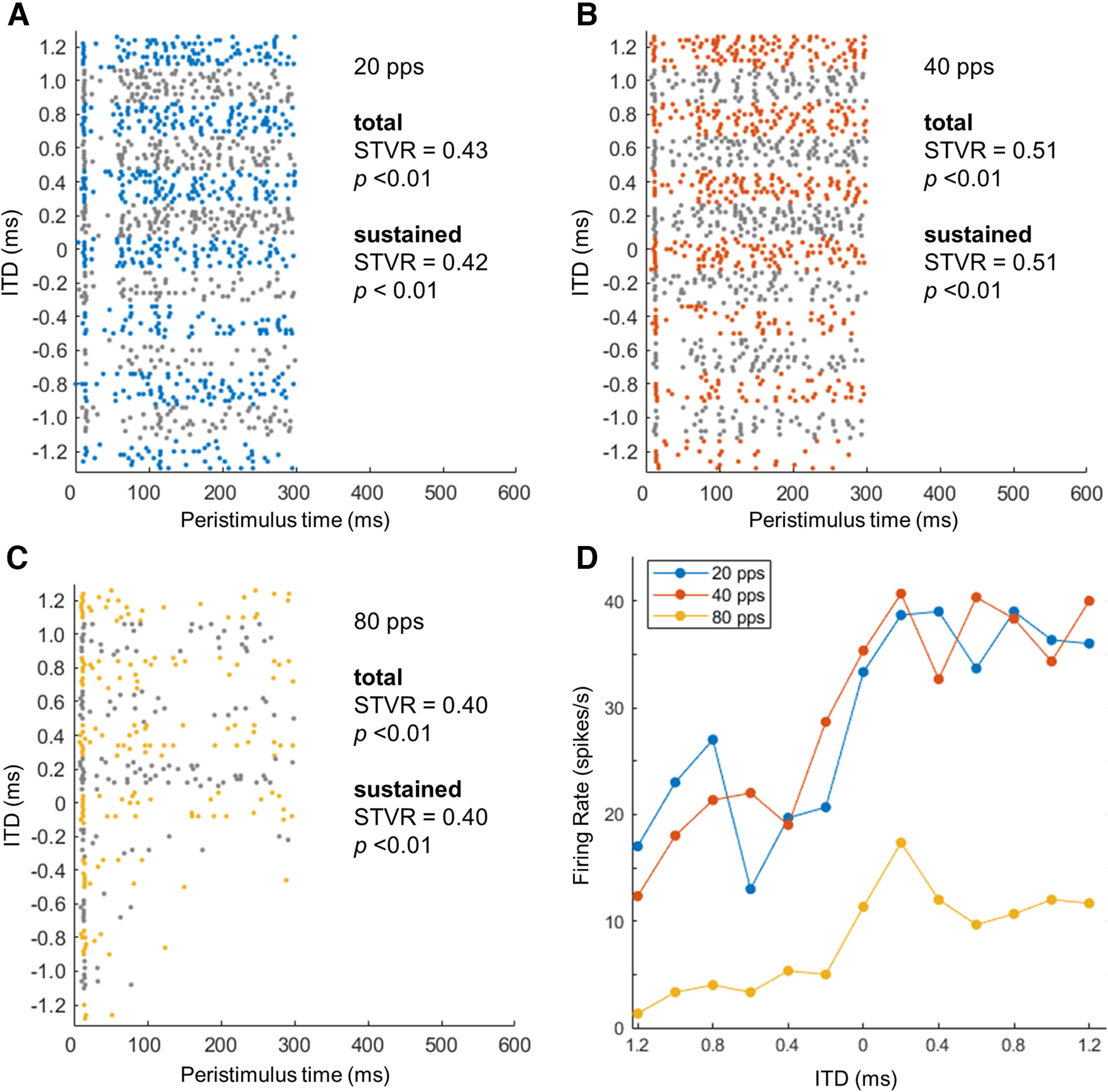
***A–C***, Temporal discharge pattern (dot rasters) to 20-, 40-, and 80-pps pulse trains as a function of the interaural time difference (ITD) for an example apical unit from a neonatal deafened rat. Signal-to-total variance ratio (STVR) values and corresponding *p*-value from the *F* test are presented for three different time segments (total responses for 0–300 ms; and sustained responses for 20–300 ms). ***D***, Firing rate of total responses versus ITD curves for a range of pulse rates for the same example neuron.

The ITD sensitivity of a single unit was estimated for each pulse rate (20, 40, 80, 160, 320, 640, and 1280 pps) by measuring the extent to which responses to 300-ms periodic pulse trains would be influenced differently according to the different levels of the ITD ranging from −1200 to 1200 μs. Figure 3 presents the neural responses obtained in a representative ITD-sensitive apical unit from an ND rat (animal ID No. 90 and unit No. 3) to pulse trains with varying ITD at various pulse rates (20, 40, and 80 pps) as raster plots and ITD tuning curves with STVR values of two different time segments (peristimulus time, 0–300 and 20–300 ms) and corresponding *p*-values from an *F* test. This example neuron showed the sustained response during 20–300 ms decreased as the pulse rate increased. Sustained responses for contralateral leading ITDs were dominant compared with those for ipsilateral leading ITDs, as indicated by the statistically significant STVR values (Fig. 3*A–C*). In particular, at a rate of 80 pps, the neuron preferred a limited range of a contralateral leading ITD, and the mean firing rate peaked around 200 μs, as shown in the ITD tuning curve (Fig. 3*D*).

Approximately 65% of the IC neurons from the ND group exhibited significant ITD sensitivity for at least two different pulse rates based on the STVR analysis (*F* test, *p* <0.01). For each stimulus location, 68.3% of the apical units and 59.5% of the basal units were sensitive to varying ITD at least two pulse rates, but this difference in the percentages of the ITD-sensitive units between the two groups did not reach statistical significance [χ*^2^*(1) = 0.792 (*n* = 100); *p *=* *0.373]

**Figure 4.**
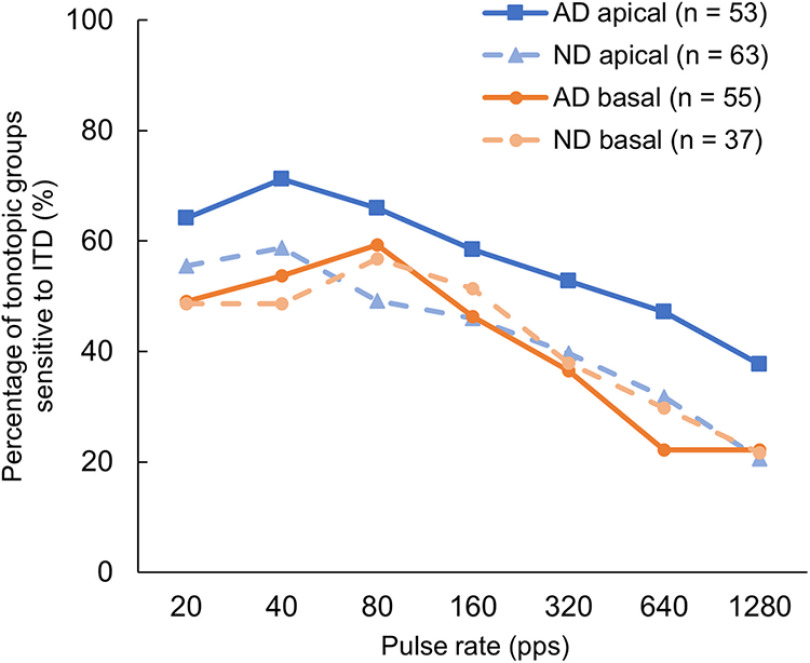
Percentage of apical and basal units with interaural time difference (ITD) sensitivity as a function of the pulse rate in acutely deafened (AD; solid lines) animals and neonatally deafened (ND; dashed lines) animals.

The percentages of the apical and basal units exhibiting statistically significant ITD sensitivity, as measured by the STVR, for each pulse rate from the different groups (AD vs ND) are shown in Figure 4, respectively. In both groups, the pulse rates with the highest percentage of ITD-sensitive neurons were 40 pps in the apical units and 80 pps in the basal units, and the percentage of ITD-sensitive units tended to decrease as the pulse rate increased. Analyses of deviance of GLMM fit for the binary variable indicating the presence or absence of ITD sensitivity were conducted in both groups. Statistical analysis showed no significant interaction effects between the pulse rate and place of stimulation in both AD and ND groups [χ^2^(6) = 3.464 (*n* = 108); *p *=* *0.749, and χ^2^(6) = 3.134 (*n* = 100); *p *=* *0.792, respectively]. In the AD group, there were significant main effects for both the pulse rate [χ^2^(6) = 52.789 (*n* = 108); *p *<* *0.001] and place of stimulation [χ^2^(1) = 31.536 (*n* = 108); *p *<* *0.001] on the percentage of ITD-sensitive neurons. However, when the same analysis was conducted with data from the ND group, the effect of the pulse rate was also significant [χ^2^(6) = 49.504 (*n* = 100); *p *<* *0.001], but the effect of the location of stimulation was no longer significant [χ^2^(1) = 0.214 (*n* = 100); *p *=* *0.644]. Then we can conclude that, in the AD group, more apical units were sensitive to varying ITDs than basal units for all pulse rates tested as shown in Figure 4 In contrast, in the ND group, the percentage of ITD-sensitive neurons was comparable between the apical and basal units at most rates.

**Figure 5.**
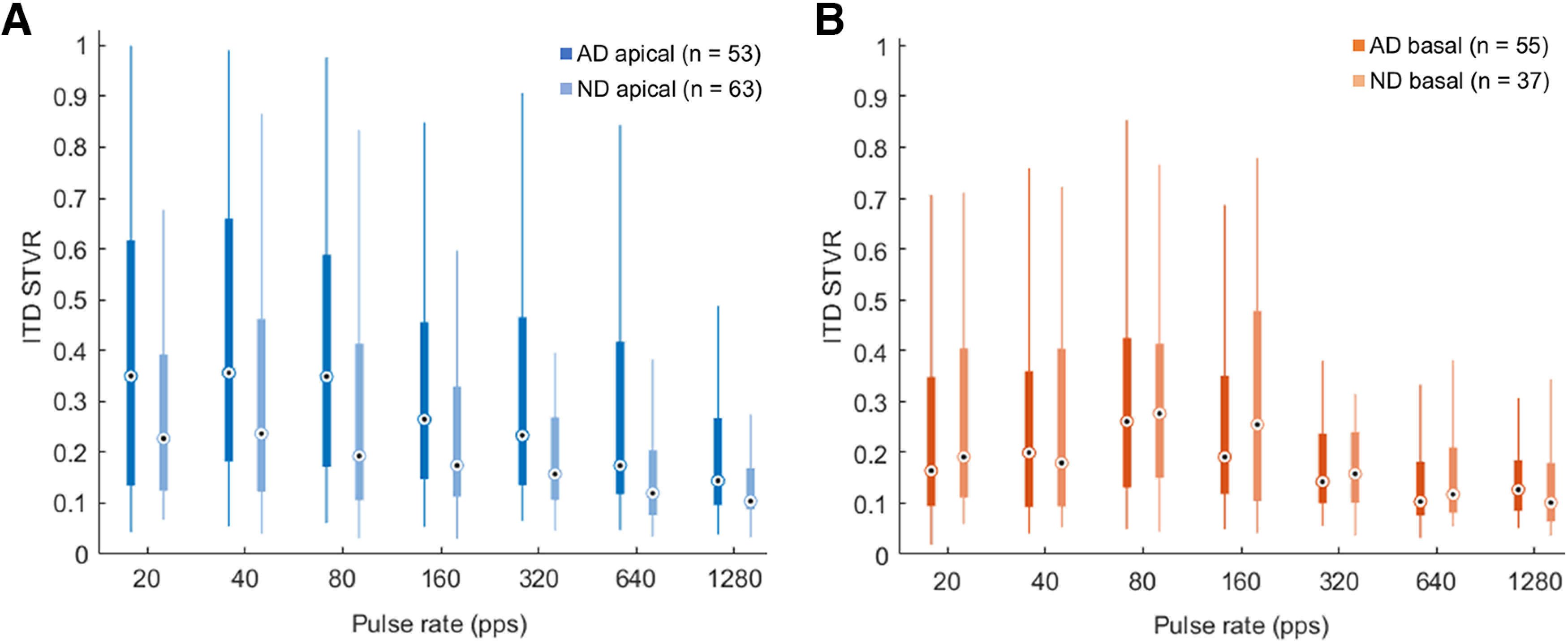
Interaural time difference (ITD) signal-to-total variance ratio (STVR) as a function of pulse rates in acutely deafened (AD) animals and neonatally deafened (ND) animals to apical (***A***) and basal pair stimulations (***B***). The central black dot inside a white circle indicates the median, and the bottom and top edges of the box present the 25th and 75th percentiles, respectively.

To compare the degree of ITD sensitivity between the AD and ND groups, a two-way ANOVA with LMMs on the logit-transformed ITD STVR was conducted for apical units (Fig. 5*A*) and basal units (Fig. 5*B*), respectively. Because ITD STVR values are nonbinomial and range between zero and one on a proportional scale by definition, a logit transform was conducted to fulfill linear modeling assumptions (Warton and Hui, 2011). The interaction effects between early auditory deprivation and pulse rate on ITD sensitivity were nonsignificant in both apical and basal units (*F*_(6,710.78)_ = 0.7539; *n* = 116; *p *=* *0.606, and *F*_(6,560.15)_ = 0.6905; *n* = 92; *p *=* *0.657, respectively). For apical units, the main effect of early auditory deprivation yielded an *F* ratio of *F*_(1,794.90)_ = 14.5309, *p *<* *0.001, indicating that being neonatally deafened animals was associated with a lower ITD STVR value compared with AD animals, for each level of pulse rates (Fig. 5*A*). However, the main effect of early auditory deprivation was not statistically significant in basal units (*F*_(1,621.41)_ = 0.0178, *p *=* *0.894), and which were similar across all seven levels of pulse rate (Fig. 5*B*).

**Figure 6.**
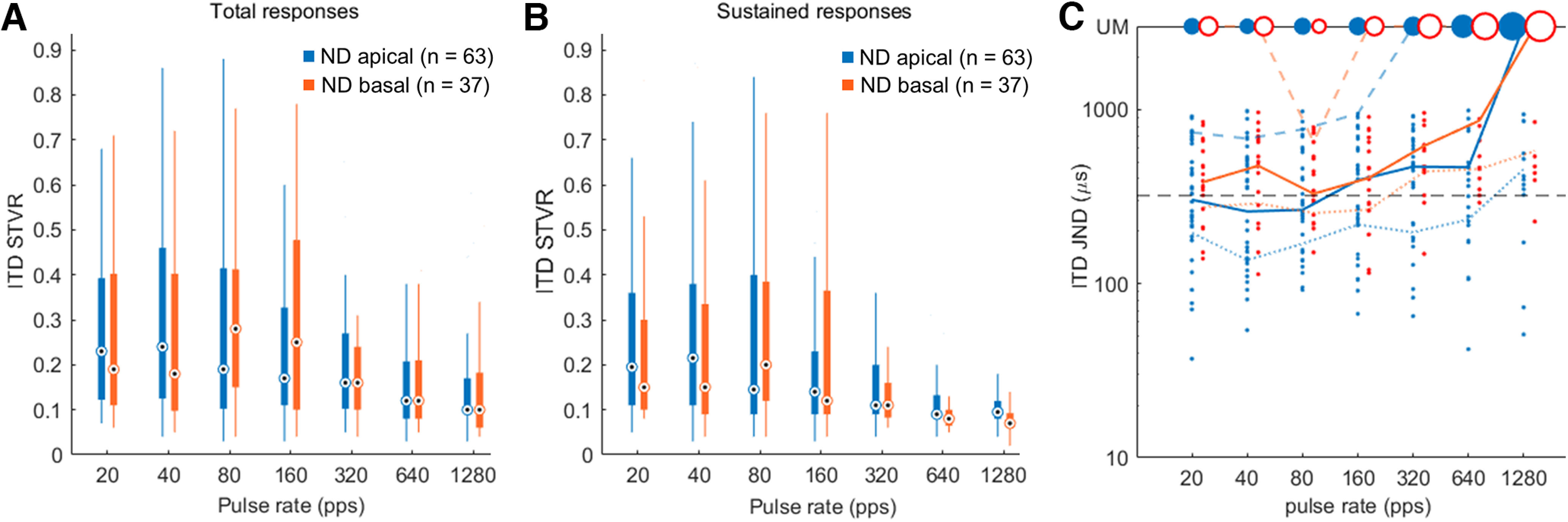
Interaural time difference (ITD) signal-to-total variance ratio (STVR) at various pulse rates for apical and basal units based on responses for different time segments, including total (0–300 ms; ***A***) and sustained (20–300 ms; ***B***), in neonatally deafened (ND) rats. The central black dot inside a white circle indicates the median, and the bottom and top edges of the box present the 25th and 75th percentiles, respectively. ***C***, ITD just noticeable difference (JND) versus the pulse rate from individual units for apical and basal stimulations. The area of the circles at the top is proportional to the number of units with unmeasurable (UM) JNDs. The dotted, solid, and dashed lines represent the 12.5th, 25th, and 50th percentiles, respectively. The horizontal black dashed line illustrates the maximum ITD difference in adult rats (320 μs).

In the ND group, ITD sensitivity was compared based on ITD STVRs and neural JNDs from the apical and basal units. Figure 6*A* shows the distribution of the STVR values for the apical and basal units across all tested pulse rates. For the logit-transformed data, a two-way ANOVA with LMMs was used to determine the effects of the cochlear location and pulse rate of electrical stimulation on the degree of ITD sensitivity. There was no statistically significant interaction between the location of stimulation and pulse rate on the ITD STVR (*F*_(6,609.91)_ = 1.086; *n* = 100; *p *=* *0.369). The main effects analysis showed that the location of stimulation did not have a statistically significant effect on ITD STVR (*p *=* *0.743), whereas the pulse rate did (*p* <0.001).

Then, a two-way ANOVA with LMMs on the logit-transformed ITD STVR based on sustained responses was repeated to evaluate the relative contribution of responses during two different poststimulus periods on the degree of ITD sensitivity (Fig. 6*B*). There was no statistically significant interaction between the pulse rate and place of stimulation (*F*_(6,566.95)_ = 1.883; *n* = 100; *p *=* *0.082). The main effect of the pulse rate on mean ITD STVR based on the sustained response was statistically significant (*F*_(6,566.97)_ = 23.6886; *n* = 100; *p *<* *0.001). Similar to total responses, the sustained STVR did not differ between the two stimulation positions (*F*_(6,590.45)_ = 0.0304; *n* = 100; *p *=* *0.862).

Figure 6*C* compares the 12.5th, 25th, and 50th percentiles (indicated by dotted, solid, and dashed lines, respectively) of neural ITD JNDs from the apical (blue) and basal (orange) units in the ND group as a function of the pulse rate. ITD JNDs could be measured for more than half of the neurons at a relatively wide range of pulse rates (20–160 pps) in apical units but only at 80 pps in basal units. All 100 units, even those with unmeasurable JNDs, were included in the percentile computations. As previously described by Chung et al. (2016), the 25th empirical quartile was chosen to compare the ITD discrimination performance, which is mainly determined by neurons with the best performance according to the lower envelope principle (Parker and Newsome, 1998). The best performance, estimated by the lower quartile of neural ITD JND data, occurred at 80 pps for both apical (265 μs) and basal units (328 μs). When considering the maximum difference in naturally possible ITD in adult rats (<320 μs; Fig. 6*C*, dashed horizontal line), poorer performance in ITD discrimination would be expected in low pulse rates conditions (20–80 pps) with basal stimulation compared with apical stimulation. However, when using a set of 10,000 bootstrap trials to estimate differences in mean 25th percentile ITD JNDs between apical and basal units at each pulse rate, differences could not reach statistical significance at any pulse rate (*p *=* *0.277 for 20 pps, *p *=* *0.140 for 40 pps, *p *=* *0.267 for 80 pps, *p* = 0.692 for 160 pps, *p *=* *0.247 for 320 pps, *p *=* *0.218 for 640pps, and *p* = 0.161 for 1280 pps).

**Figure 7.**
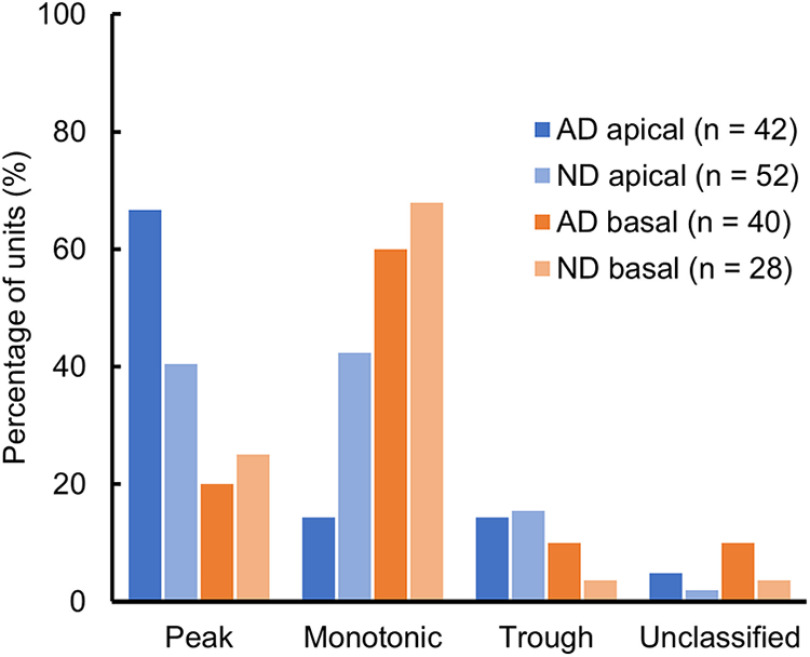
Distribution of interaural time difference (ITD) tuning types according to the location of stimulation for acutely deafened (AD) and neonatally deafened (ND) animals.

For ITD-sensitive units (80 in the ND group and 82 in the AD group) at pulse rates below 320 pps, the shape of the ITD tuning curve showing the maximum STVR value was classified into four types. Figure 7 shows the relative incidence of ITD tuning types in different animal groups according to the stimulation location. The basal units showed a similar distribution pattern between the two animal groups (*p *=* *0.568, Fisher’s exact test), and the most commonly observed classification in ITD tuning shape was monotonic, followed by a peak and then trough and unclassified types with the same proportion. However, for the units sensitive to apical stimulation, the two animal groups showed significant differences in the relative incidences of types of ITD tuning curve (*p *=* *0.011, Fisher’s exact test; with a Bonferroni correction, statistical significance was accepted at *p *<* *0.025). In the ND group, the percentage of peak-type neurons decreased and the percentage of monotonic-type neurons increased, which suggests that peak-type ITD tuning changed to the monotonic type as a consequence of neonatal deafness.

**Figure 8.**
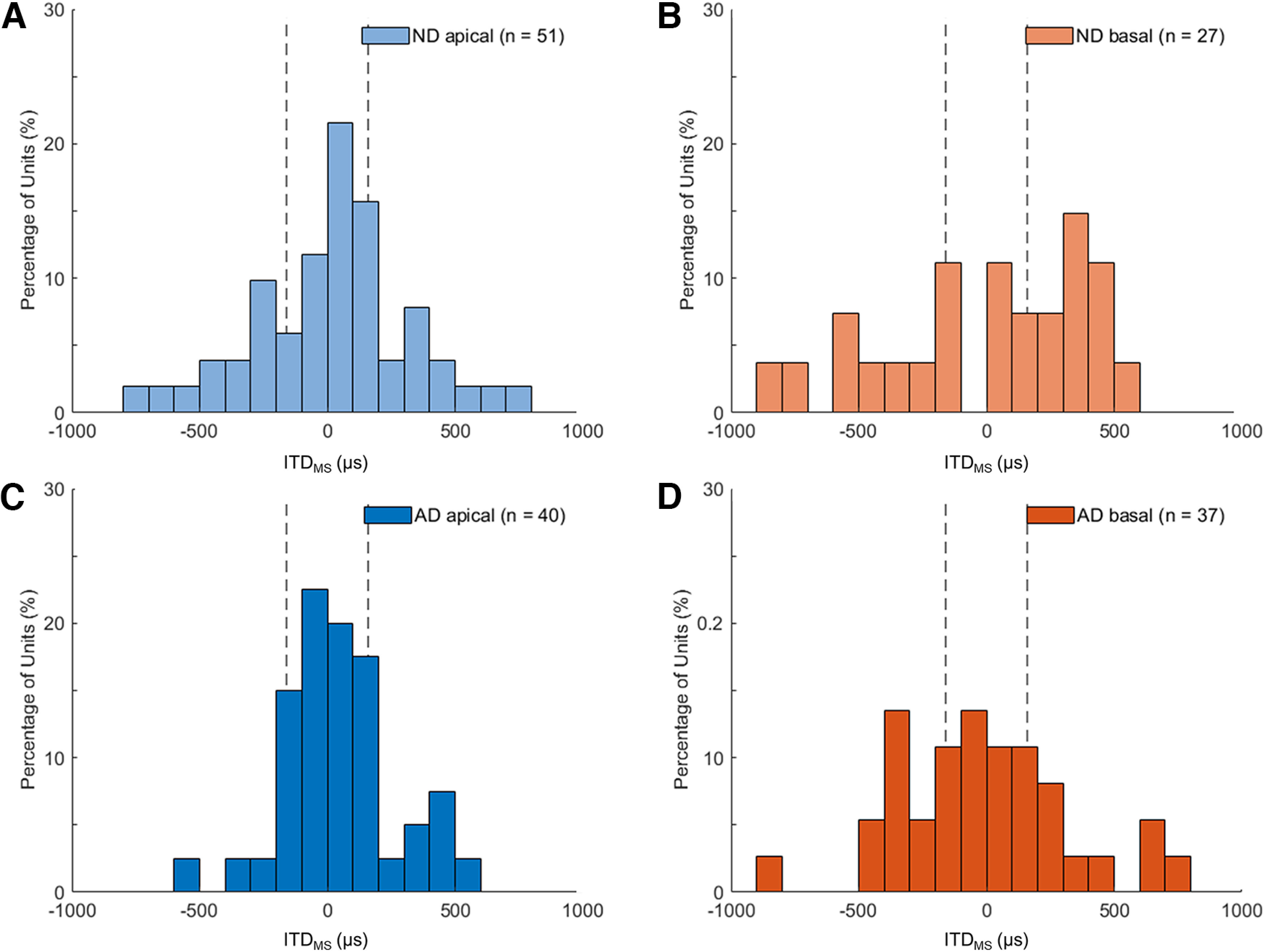
Distribution of ITD at the maximum slope (ITD_MS_) for apical (***A***, ***C***) and basal (***B***, ***D***) stimulation in neonatally deafened (ND) and acutely deafened (AD) groups. The vertical dashed lines indicate the physiological ITD range of the rat (±160 μs).

When the ITD tuning curve was classified into peak, trough, or monotonic types, we determined the ITD at the maximum slope (ITD_MS_), where the neural activity is most sensitive to ITD changes, from the fitted curve. Figure 8 shows how the distribution of ITD_MS_ differs between the apical and basal units for both groups of animals. Remarkably, similar patterns were observed in both groups of animals; the distributions of ITD_MS_ derived from apical units were significantly narrower and more concentrated around zero ITD than those from basal units (*p*-values for Levene’s test = 0.008 and 0.009 in the ND and AD groups, respectively). When comparing only apical units between the two groups, Levene’s test did not show a significant difference in the variances for ITD_MS_ (*p *=* *0.149). Consistent with the distribution of ITD JNDs in the ND group, the ND group also showed that more ITD_MS_ of apical units (45.1%) fell within the physiological ITD range (approximately ±160 μs; Fig. 8*A–D*, dotted vertical lines) than that of basal units [22.2%; χ*^2^*(1) = 3.955 (*n* = 78), *p *=* *0.047].

**Figure 9.**
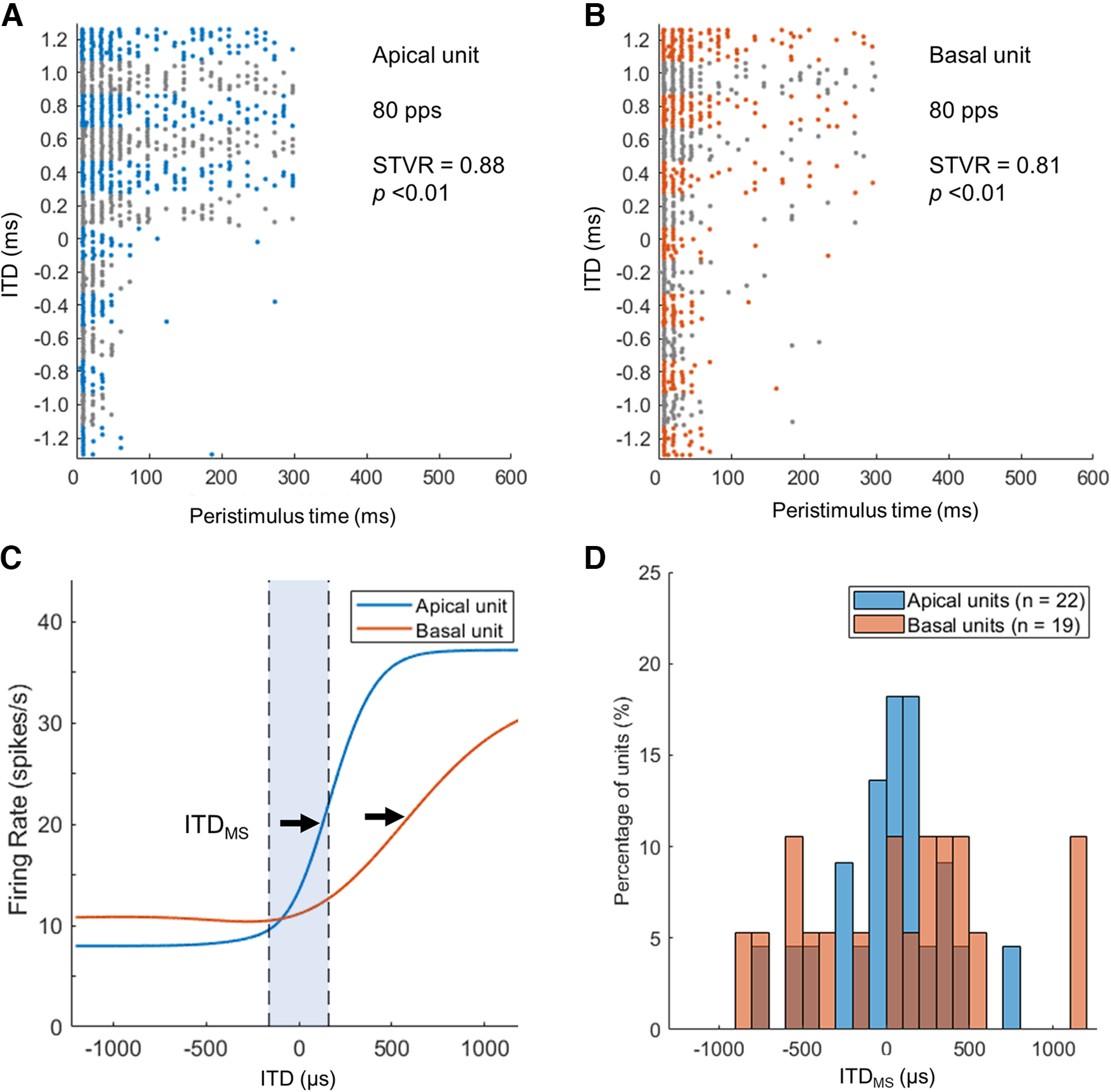
Temporal response patterns to 80 pps electrical pulse trains as a function of the interaural time difference (ITD) for one apical unit (***A***) and one basal unit (***B***). Both example neurons have a monotonic ITD tuning type and similar ITD signal-to-total variance ratio (STVR) at 80 pps. ***C***, ITD tuning curves at 80 pps for the two example neurons. The arrows indicate the maximum slope position of the ITD tuning curve. The vertical dashed lines illustrate the physiological ITD range (±160 μs). ***D***, Distributions of ITD at the maximum slope (ITD_MS_) for monotonic type units for apical and basal stimulation in neonatally deafened animals.

Thus, although apical units in ND animals showed low ITD STVR values similar to basal units, the distribution of ITD_MS_ in apical units clearly differed from those in basal units. In addition, in apical units of ND animals, the ITD_MS_ concentration within the naturally occurring ITDs was comparable to apical units of AD animals although the proportion of ITD-sensitive units with peak-type tuning was lower than that of apical units of AD animals. To explain these discrepancies, we further investigated whether there were differences in the ITD_MS_ distribution depending on tuning types between the apical and basal units (Table 1). Particularly, in ITD-sensitive units with a monotonic type of ITD tuning in the ND group, the ITD_MS_ of apical units (45.5%) tended to be concentrated within the physiological range more than twice as much as that of basal units (22.2%). The neural responses from two representative example units obtained from the ND group are presented in Figure 9*A* (apical unit) and Figure 9*B* (basal unit). Both the neurons had similar maximum ITD STVR values at 80 ps. Although both ITD-sensitive neurons had monotonic-type ITD tuning curves, they clearly showed different ITD tuning properties (Fig. 9*C*). The example apical unit was most sensitive to ITD changes at 154 μs of ITD. In contrast, the basal units were most sensitive at 563 μs, which is ∼3.5 times greater than the physiological range. Finally, Figure 9*D* presents the distributions of the ITD_MS_ calculated from the monotonic types only. In monotonic-type apical units from ND animals, the distribution of ITD_MS_ was centered closer to the midline (zero ITD) and was still significantly narrower than monotonic-typed basal units (SD 325 vs 574 μs, *p *=* *0.019, Levene’s test). In contrast, for peak-type units in the ND group, the distribution of ITD_MS_ did not differ between apical and basal units (SD 295 vs 239 μs, *p *=* *0.656, Levene’s test).

**Table 1:** Distribution of ITD_MS_ by the ITD tuning type for apical and basal stimulation in neonatally deafened animals

ITD tuning type		Percentage of units (%)			
		Peak	Monotonic	Trough	Unclassified
Apical units	ITD-sensitive units^*a*^ (*n* = 52)	40.4% (21/52)	42.3% (22/52)	15.4% (8/52)	1.9% (1/52)
	|ITD_MS_| ≤160 μs^*b*^ (*n* = 23)	38.1% (8/21)	45.5% (10/22)	62.5% (5/8)	N/A
	ITD_MS_ (μs)^*c*^	56 ± 295 (−438 to 688)	34 ± 325 (−703 to 736)	−58 ± 308 (−676 to 313)	N/A
Basal units	ITD-sensitive units^*a*^ (*n* = 28)	25.0% (7/28)	67.9% (19/28)	3.6% (1/28)	3.6% (1/28)
	|ITD_MS_| ≤ 160 μs^*b*^ (*n* = 6)	28.6% (2/7)	21.1% (4/19)	0% (0/1)	N/A
	ITD_MS (_μs)^*c*^	27 ± 239 (−222 to 332)	78 ± 574 (−801 to 1200)	454	N/A

ITD_MS_, ITD at the maximum slope.

N/A, not available to measure (see “Electrophysiological data analysis” for details).

a Only units showing significant STVR (*F* test, *p* < 0.01) with pulse rates below 320 pps.

b The physiological ITD range of the rat.

c Mean ± SD (range).

## Discussion

This study was based on single-neuronal responses to cochlear electrical stimulation as a function of ITD recorded from 100 units within the auditory midbrain of ND rats. The neural response properties were compared with data from rats with normal hearing, which were deafened during adulthood (AD). In both studies, we used site-specific penetrating intracochlear electrodes that permit frequency-specific activation in broader frequency regions than a conventional CI electrode through the round window, which makes it possible to evaluate the effect of location on ITD sensitivity more precisely. With basal stimulation to target “high-frequency” pathways, there were no significant differences in the ITD tuning between AD and ND. We found that early auditory deprivation has a major impact on patterns of ITD tuning in “apical” IC neurons which responded selectively to electrical stimulation through “low-frequency” pathways. We demonstrated that deprivation of auditory experience deteriorated the degree of ITD sensitivity, as measured using the STVR metric, in apical units to a level comparable to that in “basal” units at a “high-frequency” region. In addition, the prevalence of ITD-sensitive neurons with a peak-shaped ITD tuning curve, which may reflect predominant input from the MSO, in the ND group was diminished compared with that in the AD group. Conversely, monotonic-type responses rarely occurred in apically driven neurons of AD rats but were equally prevalent in ND rats. However, in apical units from the ND group, the distribution of ITD of the maximum slope was still more concentrated within the physiological ITD range than in basally driven units, even in the absence of binaural hearing experience.

Recent and past works demonstrating results with ITD sensitivity of single IC units in early deaf animals by use of bilateral CIs are summarized in Table 2. Above all, the greatest methodological difference between the present study and those in previous studies is that the present study achieved a very selective apical stimulation by the use of bipolar electrode pairs with one electrode in the scala tympani and the other in the scala vestibuli. Previous studies in both congenitally deaf cats (Hancock et al., 2010, 2013) and ND cats (Thompson et al., 2021) have reported an overall reduction in the incidence of ITD-sensitive neurons in the IC compared with AD cats. In contrast to the studies mentioned above in cats, we did not observe a robust decrease in the incidence of ITD sensitivity in IC neurons in ND rats. Compared with 48–53% of congenitally deaf cats or 56% of neonatally deafened cats, a higher proportion (65%) of all IC neurons recorded from ND rats showed significant ITD sensitivity. Owing to the relatively limited low-frequency hearing in rats compared with cats, species differences may account for this possible discrepancy. However, a previous study using ND rabbits, a species with the similar low-frequency hearing ability and neural tuning to ITDs as cats (Heffner and Masterton, 1980; Ebert et al., 2008), reported a similar proportion (62%) of ITD-sensitive neurons to our findings (Chung et al., 2019). In addition to species differences, there are significant methodological differences, considering that previous studies in cats mainly used low-rate pulse trains (10–80 pps), whereas the study with rabbits and ours used a pulse train with a wide range of pulse rates between 20 and 1280 pps. Thus, when observing only data obtained using a 40-pps pulse train (same as Thompson, et al., 2021), nearly half of the IC neurons showed ITD sensitivity in ND rabbits compared with AD rabbits (Chung et al., 2019). Similarly, in our ND rats, the overall incidence of ITD-sensitive neurons at 40 pps was markedly reduced to 55%, which fell within a similar range (48–56%), as reported in previous studies on cats. In particular, in cases of basal stimulation, more than half of the neurons were insensitive to ITD changes under low pulse rate conditions (20–40 pps).

**Table 2:** Comparison with other published works

	Hancock et al. (2010)	Hancock et al. (2013)	Thompson et al. (2021)	Chung et al. (2019)	This work
Species	Cats	Cats	Cats	Rabbits	Rats
Deafening procedure	Congenitally deaf	Congenitally deaf	Neonatal deafening (neomycin sulfate)	Neonatal deafening (neomycin sulfate)	Neonatal deafening (kanamycin)
Approaches for implant insertion	Round window	Round window	Round window	Round window	Cochleostomies
Electrodes	8-ring electrode array	8-ring electrode array	8- or 14- ring electrode array	8-ring electrode array	4 independent electrodes
Stimulus configuration	Wide bipolar^*a*^	Wide bipolar^*a*^	Wide bipolar^*a*^	Wide bipolar^*a*^	Scala tympani and scala vestibuli electrodes pair
Pulse rates (pps)	10–80	10–80	40	20–1280	20–1280
Phase duration and interphase gap (μs)	50/0	50/0	100/50	50/0	40/0
Analysis metric of ITD sensitivity	STVR (*F* test, *p* < 0.025)	STVR (*F* test, *p* < 0.025)	STVR (*F* test, *p* < 0.025)	STVR (*F* test, *p* < 0.01)	STVR (*F* test, *p* < 0.01)
Incidence of ITD sensitivity (no. of units)	48% (114)	53% (139)	56% (55)	62% (66)	65% (100) 55% (100)^*b*^

STVR, the signal-to-total variance ratio.

a Between the most apical and most basal intracochlear electrodes.

b Only at a pulse rate of 40 pps.

We did not find robust differences in the degree of ITD sensitivity based on the ITD STVR between the apical and basal units in the ND group. This lack of a robust place effect contrasts with a previous study using AD rats with normal hearing experience, which reported significantly higher ITD STVRs for pulse rates of 20, 40, 80, and 320 pps in apical units compared with those in basal units (Sunwoo and Oh, 2022). Previously, we also found that ITD sensitivity to apical stimulation was determined predominantly based on the sustained response at low pulse rates (20–80 pps) in AD rats, while ITD STVRs based on the onset response were better than sustained STVRs at high pulse rates (320 and 1280 pps). This is consistent with the results in AD cats showing that ITD JNDs in IC neurons are dominated by sustained responses at lower pulse rates (40–80 pps), whereas onset responses mostly contribute to ITD JNDs at higher pulse rates (160–320 pps; Smith and Delgutte, 2007). Chung et al. (2019) also reported a reduction in the prevalence of synchronized responses and a reduction in the strength of synchrony, particularly at low pulse rates, in rabbit IC neurons following neonatal deafness. It has been demonstrated that the most likely anatomic substrate for precise phase locking of IC neurons to the fine structure of electrical pulse trains is the brainstem pathway from the spherical bushy cells in the cochlear nucleus to principal cells of the MSO and IC neurons in the low-frequency region corresponding to apical units in the current study (Middlebrooks and Snyder, 2010). Considering this pathway, the peak type ITD tuning curve was observed most prominently in the apical units of AD rats, as expected from the tuning of MSO neurons, which showed the highest responses to binaural stimulation at favorable ITDs. However, in the apical units of ND rats, the prevalence of peak type tuning was fairly low (40% vs 67% in AD rats), and approximately equal to the sigmoid type (42% vs 14% in AD rats), which is similar to the typical function of high-frequency LSO neurons, with the convergence of ipsilateral inhibitory and contralateral excitatory inputs (Caird and Klinke, 1983). Our findings indicate that early auditory deprivation can impair sensitivity to the temporal fine structure of electrical pulse trains through the low-frequency brainstem pathway and result in more degradation in neural ITD sensitivity at low than at high pulse rates.

Previous studies on bilateral CI users with prelingual or early onset deafness suggest an important role of early auditory input in the establishment of ITD sensitivity (Litovsky et al., 2010, 2012; Salloum et al., 2010). Patients with early auditory deprivation showed no measurable sensitivity to ITDs on the lateralization task, whereas recent studies in rats have shown a comparable behavioral ITD sensitivity between animals with NH and those who were ND and received bilateral CIs (K. Li et al., 2019; Rosskothen-Kuhl et al., 2021). It should be noted that Rosskothen-Kuhl and colleagues, fully inserted the animal electrode array into the middle turn through a cochleostomy and not into the basal turn via the round window, as performed in human studies. In addition, it is possible that there was less interaction between the target auditory neurons of active electrodes and neighboring neurons activated by electrical current spread when a narrow bipolar stimulation mode (two most apical channels) with relatively low current intensity (≤300 μA) was used so that high behavioral sensitivity to ITDs could be achieved compared with human data measured by monopolar stimulation, even with much higher current intensity. In the same context as reducing the spread of excitation, a noticeable trend in ITD thresholds across tonotopic frequencies was observed in neural ITD JNDs in AD rats (Sunwoo and Oh, 2022). Despite no significant differences in ITD STVRs and JNDs between apical and basal units in ND rats, high ITD discrimination performance to apical stimulation could be possible based on the clearly distinct distribution of ITD_MS_ observed in the ITD-sensitive neurons, as presented in Figure 8*A*,*B*. Although neonatal deafening affected the distribution of ITD tuning types in the majority of apically driven IC units, early auditory deprivation during development had a minimal effect on the distribution of ITD_MS_. Hancock et al. (2013) reported that the ITD tuning curve often changed from a peak type to a monotonic type (“sigmoid” in their study) as the stimulus level increased in both acutely and congenitally deaf cats. Furthermore, it was observed that the ITD STVR remained stable despite the changes in the types of tuning curves with increasing levels. Based on these findings, we can speculate that the increase in the prevalence of monotonic-typed ITD tuning curves in apical units in the ND group compared with the AD group is more likely because of the level dependence changes rather than impairment of the basic brainstem pathways that are functionally segregated by frequencies in normal hearing animals. These outcomes further support our hypothesis that selective stimulation of low-frequency pathways is an important factor that affects the improvement of ITD sensitivity in bilateral CI users, even after early auditory deprivation. However, how well ITD sensitivity might be improved by selective stimulation of apical regions when psychophysically measured in this animal model remains to be tested in future studies.

## Synthesis

Reviewing Editor: David Schoppik, New York University Decisions are customarily a result of the Reviewing Editor and the peer reviewers coming together and discussing their recommendations until a consensus is reached. When revisions are invited, a fact-based synthesis statement explaining their decision and outlining what is needed to prepare a revision will be listed below. The following reviewer(s) agreed to reveal their identity: John Middlebrooks, Mitchell Day. Note: If this manuscript was transferred from JNeurosci and a decision was made to accept the manuscript without peer review, a brief statement to this effect will instead be what is listed below.

Both Reviewers felt that while the manuscript advanced the field, they had reservations about the writing. They felt that the grammatical errors detracted from the data presentation and we recommend having the revision either proofread professionally or using software such as Grammarly. The bulk of the “minor” comments below highlight areas where presentation could be clear.

Major concerns

1) Neuronal ITD sensitivity (as captured by the ITD STVR metric) is not directly compared between neonatally and acutely deafened animals. Statistical comparisons were made regarding proportion of ITD-sensitive neurons, but such a comparison does not reveal whether ITD-sensitive neurons are STRONGLY or WEAKLY ITD-sensitive-a comparison of ITD STVR’s would show this and is therefore important.

2) The statistical analyses of onset responses have very low statistical power due to low number of stimulus repetitions. Most results in the study involve firing rates over the stimulus duration (300 ms) averaged over 10 repetitions. To get a sense of the accuracy of firing rate estimation, assume spike count follows a Poisson distribution (mean = variance) and take a neuron that fires at 50 spike/sec over the stimulus duration (= 15 spikes, on average). For 10 stimulus repetitions, the standard error of the estimate of mean spike count would be stand dev/sqrt(N) = sqrt(15)/sqrt(10) = 1.2 spikes (4 spikes/sec), which is reasonably good precision for a 50 spikes/sec rate. However, a 50 spike/sec rate over the onset time window (20 ms) would have a standard error of sqrt(1)/sqrt(10) = 0.32 spikes (16 spikes/sec), which is very poor estimation precision. Good estimation of firing rates over smaller time windows requires more stimulus repetitions. It is no surprise that ITD STVRs of onset responses are very low because the estimates of onset firing rates are full of estimation error. Since onset responses are not estimated accurately, they should not be reported.

3) ITD JND’s: First, the text regarding Figure 4D states that only the lowest quartile of data is shown. Results are interpreted based on the median of that data. The median of the lowest quartile is the 12.5th percentile, by definition. The use of the 12.5th percentile in interpreting the data is arbitrary-one could use the 25th percentile or the 10th percentile and come to different conclusions. I understand that comparing the distributions is of interest. Perhaps it would be better to show and interpret the data from a set of percentiles (e.g., 10th, 20th, and 30th) instead of only the 12.5th percentile. Second, the description of the ITD JND metric in the Methods is unclear. Please include an illustration using real data. What does it mean that JND’s were computed for reference ITDs from -160 to +160 microsec when ITD sampling was in 200-microsec steps? Since you are wanting to examine the physiological ITD range, why not fix the reference ITD at zero? Was the metric computed from spike counts or firing rates? (Should be spike counts.) Methods state that JND was computed by dividing by “the arithmetic mean of variances”, but this should be the square root of the arithmetic mean of variances, according to the author’s previous paper. Best to list the equation.

4) The author states in many places (including the Abstract) that the prevalence of peak-type ITD tuning curves suggests input from medial superior olive onto IC neurons. In the Discussion (lines 520-1), they erroneously state that sigmoid-shaped ITD tuning curves are typical of lateral superior olive neurons. They are getting binaural cues mixed up here. Lateral superior olive neurons have sigmoid-shaped ILD tuning curves, not ITD. Their ITD tuning curves are trough-type due to the coincidence of excitation and inhibition. The author puts a lot of emphasis on the change in proportion of peak-type vs. sigmoid-shaped ITD tuning curves with postnatal deafness, likely because that is one of the few differences observed, but there is no reason to believe there are differences in origin of the ITD sensitivity underlying peak-type and sigmoid-shaped sensitivity.

Minor / line-by-line

The meaning of the title is not clear to me. Prior to reading the paper, I guessed that it means that peak and trough sensitivity to ITD became somewhat but not entirely more similar after deprivation. After reading the paper, I see that deprivation resulted in a decrease in peak responses and an increase in monotonic responses among apical units but had minimal effect on basal responses. I cannot see how that indicates partial maintenance of parallel pathways. Indeed, I still don’t even know what the authors mean by parallel pathways. Is it apical/basal or peak/monotonic? Also, “auditory deprivation” seems like it might mean rearing in a quite environment or a conductive hearing loss, whereas what was really done was profound ototoxic sensorineural deafness. I think that it would be better for the title to emphasize that neonatal deafening selectively degrades ITD sensitivity in low-frequency pathways.

- The Abstract doesn’t mention the term “cochlear implant”, which makes it confusing to understand why the study uses electrical stimulation. Should be mentioned within the first few sentences.

- The use of the terms “low-frequency neurons” and “high-frequency neurons” throughout the manuscript assumes too much since auditory stimuli are not used in this study. Better to use terms such as “apically driven neurons” or “basally driven neurons”.

Lines 33-34: “with ITDs delivered at two intracochlear locations representing the maximum difference in their tonotopic axes” seems to indicate ITD stimuli consisting of an apical stimulus in one ear and basal stimulus in the other, which certainly is not what was done. Please re-write here and elsewhere: perhaps say that electrical stimuli were delivered to the apical or basal cochlea.

Lines 35-36: Please indicate here that the “major impact on the ITD tuning patterns” was “restricted to” low-frequency IC neurons. Then, insert “low-frequency” in “the prevalence of “low-frequency” ITD-sensitive neurons with a peak-shaped ITD curve...”

Lines 38-39: This is difficult to follow. In line 38, you say that the prevalence was diminished and then give a pair of numbers that increase (40% and 67%) and you name the experimental group (ND) prior to the control (AD). In the next sentence, you say AD prior to ND, but give the numbers as ND (42%) prior to AD (14%). I suggest that you consistently give the control number and group name (AD) prior to the experimental number and group name (ND).

Lines 44-46: Again, I don’t see what is meant by “segregated pathways are partly maintained”. Do you mean that the apical pathway is largely degraded but the basal pathway is maintained?

Lines 61-62: “patients, with limited benefit” should be “patients who have limited benefit” (with no comma).

Line 73 “can ITD” seems to be missing a word.

Lines 85-87: Neither of the two cited papers said anything about electrical stimulation or cochlear implants.

Lines 105-106: Again, “ITDs delivered at two intracochlear locations representing the maximum difference in their tonotopic axes” is misleading.

Line 119: Should say (I think) that the AD rat were deafened on the same day as they were implanted.

Figure 1A: Please indicate in the figure the approximate locations of the electrodes.

Figure 1B: It also looks like the apical area in the ND example is decreased. Was this a general finding or just the orientation of this particular section? May be interesting to compare area in addition to cell density.

Lines 161-4: It it really the “same?” It is important to state that rats were implanted unilaterally (not bilaterally) in the pilot study, which was how you determine frequency tuning using auditory stimulation of the intact ear.

Lines 181-182: Please give the range of eABR thresholds that was encountered.

Line 198: “reactive neurons” should be “responsive neurons”.

Lines 244-247: This is unclear. I take it to mean that you computed the standard separation for successive pairs of ITD values and selected as the JND the smallest increment in ITD that gave a separation larger in magnitude than one. Please clarify that. Also, lines 246 and 247 seem to say that an ITD JND was considered to be measureable only if the magnitude of the standard separation was >1 at all ITD increments, which I think is not what is intended.

Line 252, “First”: This seems to say that the first step in processing was to classify the shape of the ITD curves, whereas it seems like the first step was to compute the curve, which is given in the previous sentence. It would be helpful to begin this paragraph with a description of peak, monotonic, and trough ITD curves.

Lines 332-334: It is not clear what was tested here by the Wilcoxon test. Is the point that rats showed a difference between apical and basal SGN density in the ND group but not the AD group? Or, that AD versus ND showed a difference among apical but not basal SGNs? Please clarify.

Line 347 I think that “at all three” should be “at any of the three”.

Line 355: What test or experimental groups does p<0.01 refer to?

Figure 3 and relevant text: Lines 359 and 360 say what I think is really meant, that the plots represent the fraction of apical and basal neurons that are ITD sensitive. The vertical axis label and figure legend and further text, however, refer to the “Fraction of ITD sensitive units”, which seems to indicate the fractions of all ITD sensitive units that are apical or basal. That means that the values plotted for apical and basal at each pulse rate should sum to 100%, which is not what is seen. Also, the axis plots percentages, not fractions. I think that the vertical axis label should be something like “Percentage of tonotopic groups sensitive to ITD”.

In Figure 3, I would have liked to make direct comparisons of apical units in ND-versus-AD conditions and basal units in ND-versus-AD conditions. Perhaps the authors could plot all 4 conditions on the same axes, using different line types for ND versus AD.

Lines 397-399: “Both the onset-STVR and sustained-STVR did not differ...at all tested pulse rates” should read “Neither the onset-STVR nor the sustained-STVR differed...at any tested pulse rate.” Was ITD STVR computed from firing rates or spike counts? (Should be spike counts.)

Lines 417-418: “in the two animal groups” should say “between the two animal groups”.

Lines 423-427: The order of presentation here is not effective. It starts with “the most common ITD classification [in the ND group} was monotonic”, which is interesting only because monotonic responses were uncommon in the AD group. I think that it would be more straightforward to state that, in the ND group, the percentage of peak neurons decreased and the percentage of monotonic neurons increased, suggesting that peak-type tuning changed to monotonic as a consequence of neonatal deafening.

Line 460 “ND.” should be “ND rats.”

Line 465: It would be helpful to begin here with a statement that there were no significant differences in the ITD tuning of basal neurons between AD and ND. Then, follow with the interesting changes that were found only among the ND neurons.

Lines 478-497: I think that the greatest methodological difference between the present study and those in the literature is that the present study achieved a very selective apical stimulation by use of bipolar electrode pairs with one electrode in the scala tympany and the other in the scala vestibuli. That certainly should be mentioned here in this comparison with past results. The large number of percentages in this paragraph are difficult to keep track of. It would be helpful to put them in a table.

Line 533: It is not clear what is meant by “less interaction”. Do you mean less interaction between tonotopic loci? Please clarify.

Line 538 “across frequencies” could be taken to mean across tonotopic frequencies or across pulse rates. Please clarify.

Line 689, Figure 2D legend: Please indicate that these are (I think) sustained firing rates.

Methods:

- Please state the age range of animals in the AD group.

- Please include more methods on measurement of ABRs.

- The Methods state that single-unit recordings were made and gave acceptable methods demonstrating cluster analysis was performed. However, please add a criterion of small percentage of submillisecond interspike intervals to claim that recordings are single-unit.

- Please clarify why shape analysis of ITD tuning curves was only performed on data with pulse rates < 320 pps.
